# Synthesis of 1,4-Bis(phenylethynyl)benzenes and Their Application as Blue Phase Liquid Crystal Composition

**DOI:** 10.3390/ijms141223257

**Published:** 2013-11-25

**Authors:** Ning Li, Zhengqiang Li, Xing Zhang, Ruimao Hua

**Affiliations:** 1 Department of Chemistry, Tsinghua University, Beijing 100084, China; 2 Beijing R&D Center, Shijiazhuang Chengzhi Yonghua Display Materials Co. Ltd., Beijing 100083, China; E-Mails: nnl3875@163.com (N.L.); zhengqiang9527@gmail.com (Z.L.); zx@slichem.com (X.Z.); 3 Hebei Engineering & Technology Center for FPD Materials, Shijiazhuang 050091, China

**Keywords:** 1,4-bis(phenylethynyl)benzene derivatives, blue phase, liquid crystal

## Abstract

A number of 1,4-bis(phenylethynyl)benzene derivatives (BPEBs) and their analogues with different numbers of side-substitute fluorine atoms on benzene rings, and alkyl chains, ethoxyl groups, fluorine atoms and trifluoromethyl groups as the end groups have been synthesized. The effects of the different substituents on their properties such as thermal behavior of melting point and clearing point, the temperature of nematic phase, optical anisotropy and dielectric anisotropy have been well investigated, and it has been found that some BPEBs have a wide range of the nematic phase temperature with high optical anisotropy (Δ*n*) and acceptable dielectric anisotropy (Δɛ), which have been applied as the crucial compositions to constitute a liquid crystal mixture having the properties of Δɛ = 29.0 and Δ*n* = 0.283 at 25 °C. With the addition of the chiral dopant to the obtained liquid crystal mixture, blue phase liquid crystal with a blue phase temperature range of 8 °C has been achieved.

## 1. Introduction

Liquid crystals (LCs) have had a multitude of applications in the past few decades [1,2]. One of the important and unique applications is their use as the key fundamental materials to develop LC displays [3], which have actually changed people’s lifestyle due to the use of mobiles, notebook computers, flat panel desktop monitors, and LCD televisions, *etc*. The development of excellent LC displays, which have the advantages of fast response, high contrast ratio, and low driving voltage, depends greatly on the development of new types and properties of LCs. The demand for LCDs with a fast response is one of the crucial factors to improve the quality of the displays, and blue phase liquid crystal (BPLC) is commonly considered to be one of the strongest candidates [4–7].

**Scheme 1.**
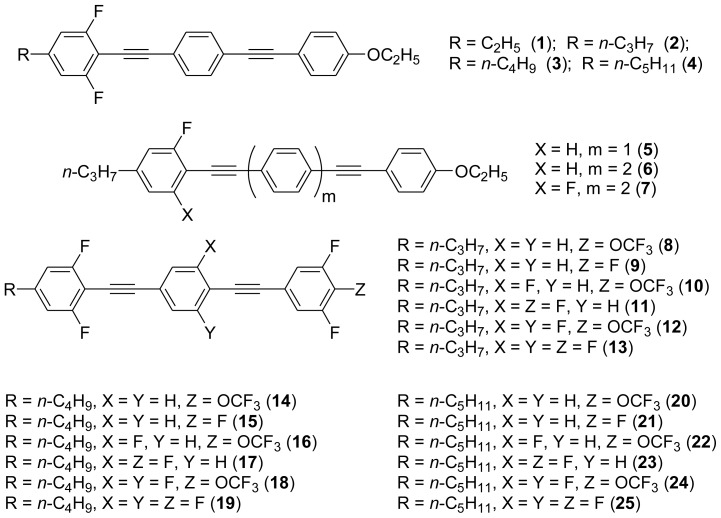
The structures of 1,4-bis(phenylethynyl)benzene derivatives and analogues (**1**–**25**).

On the other hand, the molecular and electronic structures of 1,4-bis(phenylethynyl)-benzene derivatives (BPEBs), as well as their applications have attracted much attention recently [8–12]. In particular, BPEBs have been applied as the important components in LCs with the characters of high melting point (mp), clearing point (cp), and large optical anisotropy values (Δ*n*) [13–22]. It is important and interesting to investigate the substituent effect on the properties of BPEBs. Therefore, in this paper, we describe the synthesis of a number of BPEBs with different alkyl chains, the numbers of fluorine atoms and other substituents in benzene rings as shown in Scheme 1, and the study on the substituent effects on their properties, as well as their application as blue phase liquid crystal composition.

## 2. Results and Discussion

### 2.1. Synthesis of BPEBs and Analogues (**1**–**25**)

**Scheme 2.**
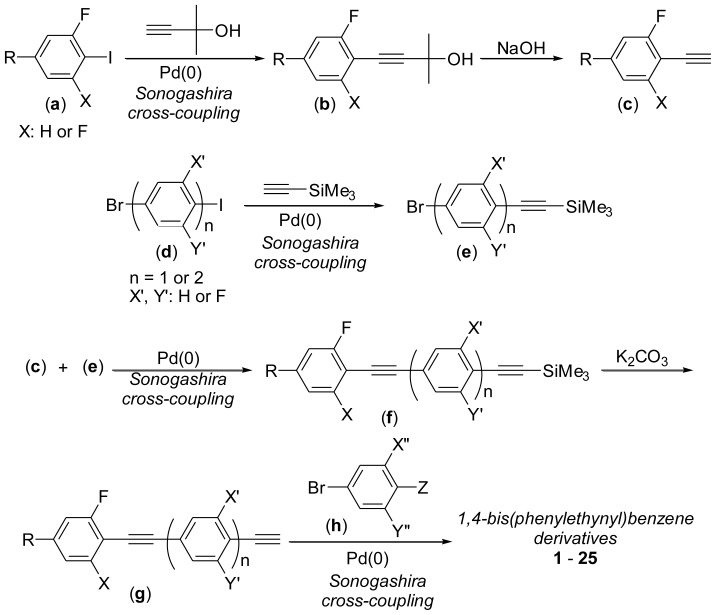
Synthesis of 1,4-bis(phenylethynyl)benzene derivatives and analogues.

The synthetic routes of **1**–**25** are outlined in Scheme 2, including the key steps of the formation of terminal and internal alkynes via Sonogashira cross-coupling reactions of aryl iodides/bromides catalyzed by palladium(0) complexes in good to high yields, and a typical synthetic procedure for the formation of **12** is described in the Experimental Section (*vide infra*).

### 2.2. Thermal Properties

The thermal properties of the melting point (mp) and clearing point (cp) are critical in the practical utilization of the synthesized BPEBs as the composition of LCs, thus the mp and cp were determined by DSC (Differential Scanning Calorimetry), and their thermal data as well as enthalpic data (Δ*H*) are concluded in Table 1. It was found that mp and cp greatly depended upon the molecular and electronic structures of BPEBs. BPEBs **1**–**4** clearly show the effect of alkyl chain length on mp and cp, and when *n*-C_3_H_7_ and C_2_H_5_ groups are used as the end groups, BPEBs **1** and **2** give the similar mp and cp (**1***vs.***2**). However, when the longer alkyl chains of C4 and C5 were employed, BPEBs **3** and **4** showed similar thermal properties, but both mp and cp decreased greatly (**1** & **2***vs.***3** & **4**) [23]. It can be concluded that BPEBs **3** and **4** bearing the longer alkyl chains have a wider range of nematic phase temperature than those of BPEBs **1** and **2**, possibly due to the longer alkyl chains being more flexible than short alkyl chains. Both **3** and **4** show a nematic phase temperature range of about 140 °C, and the wide nematic phase temperature are very important to make a practical LC mixture.

By comparison of **2** and **5**, it was found that decreasing the number of fluorine atoms in the end benzene ring leads to the increase of both mp and cp in a range of about 10 °C, and a similar trend of mp was also found between 4,4′-bis(phenylethynyl)biphenyls **6** and **7**, which are the analogues of BPEBs **2** or **5**.

**Figure 1.**
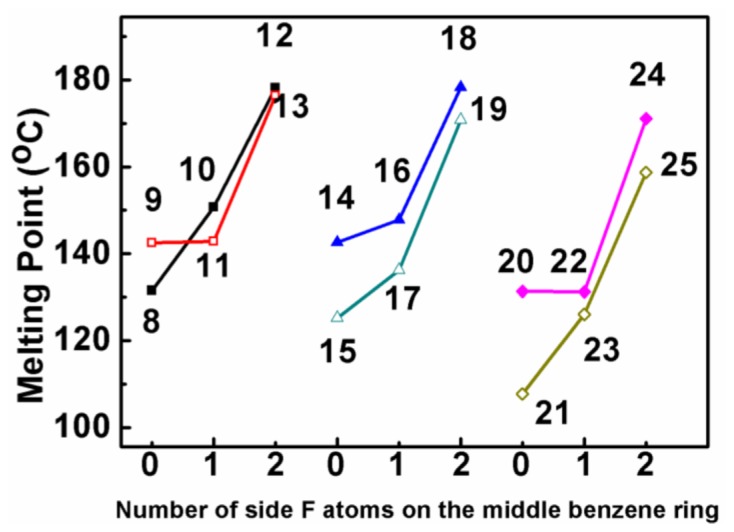
Effect of side-substituted fluorine atoms and end groups on melting points.

The thermal properties of other BPEBs are also compared to each other, as shown in Figure 1, and two notable features of the relationship between mps and their chemical structure could be concluded as follows: (1) The introduction of the fluorine atom on the middle benzene ring shows a great effect on the change of mp. In general, one or two fluorine atom-substituted benzene result in the increase of mp, and the introduction of the second fluorine atom leads to much more significant increase of mp relative to the first fluorine atom introduction (e.g., **8**→**10**→**12; 9**→**11**→**13**). (2) When the end group of OCF_3_ is replaced by a fluorine atom, the mps decreased (e.g., **10***vs.***11; 12***vs.***13; 14***vs.***15**), and only one exception (**8***vs.***9**) was observed.

### 2.3. Nematic Phase

**Figure 2.**
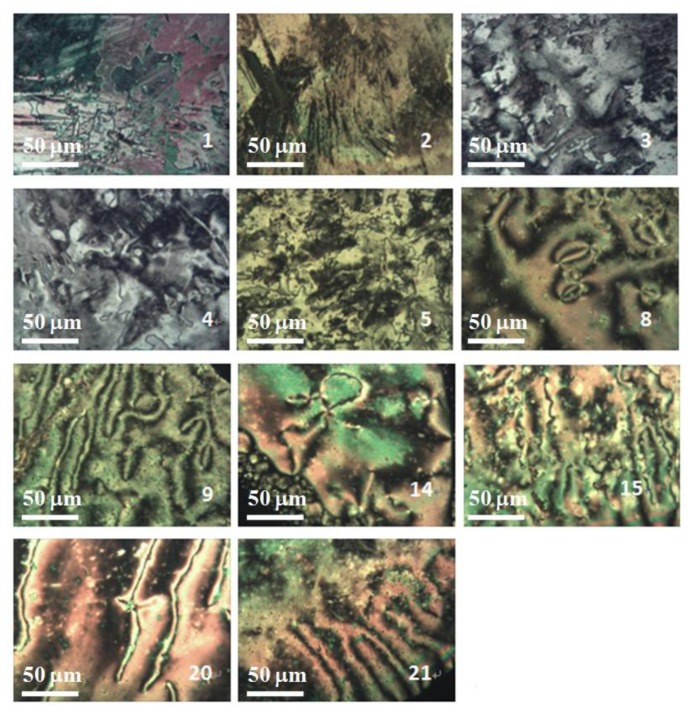
Polarizing optical micrographs photos of BPEBs **1**–**5**, **8**, **9**, **14**, **15**, **20** and **21**.

**Figure 3.**
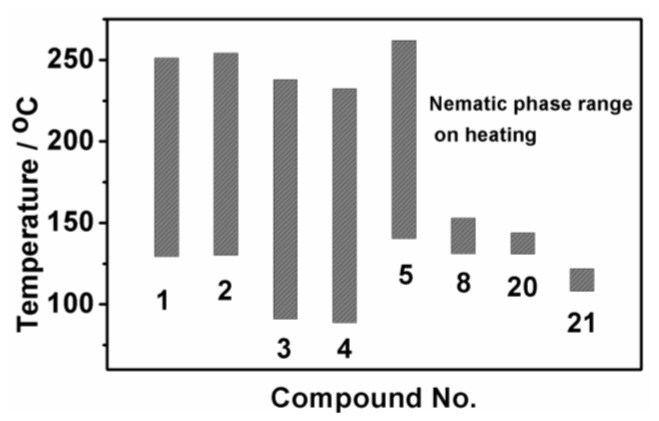
Nematic phase temperature range.

Among the synthesis of BPEBs and analogues, only BPEBs **1**–**5**, **8**, **20**–**21** (Figure 2, on heating run) and **9**, **14**–**15** (on cooling run) show nematic phases under polarizing microscope with the structural character without side-substituted fluorine atoms bonded to the middle benzene ring, and the analogues of BPEBs **6** and **7** have no nematic phase either. It was found that the nematic phase temperature is quite different depending on the end groups and the numbers of fluorine atoms. As shown in Figure 3 and Table 1, BPEBs **1**–**5** with OC_2_H_5_ as the end group possess wide nematic phase temperatures from 121.6 to 147.0 °C, and have apparently disclosed that longer alkyl chains generally result in a wider temperature of nematic phase. BPEBs **3** and **4** bearing the longer alkyl chain of *n*-C_4_H_9_ or *n*-C_5_H_11_ give the maximum nematic phase temperatures, while BPEBs **8**, **20** and **21** with OCF_3_ or F as the end groups show the relatively narrow nematic phase temperature range.

### 2.4. Optical Anisotropy (Δ*n*)

The optical anisotropy or birefringence (Δ*n*) of the BPEBs showing nematic phase in host LC was determined, and the obtained results were concluded in Table 2. As expected, a large π-conjugated structure leads to relatively high Δ*n* value, and therefore BPEBs **1**–**5** have higher Δ*n* values than **8**–**9**, **14**–**15**, and **20**–**21**. It is reasonable to understand that much more side-substituted fluorine atoms in different benzene rings will decrease the integrity of π-conjugated structures to result in a decrease of optical anisotropy.

### 2.5. Dielectric Anisotropy (Δɛ)

Table 3 shows the values of dielectric anisotropy (Δɛ) of the BPEBs having nematic phase in host LC. It was found that with the number increase of side-substituted fluorine atoms, BPEBs **8**–**9**, **14**–**15**, and **20**–**21** have higher dielectric anisotropy values than BPEBs **1**–**5**. In addition, comparison of BPEBs with the end groups of OCF_3_ (**8**, **14**, **20**) and F (**9**, **15**, **21**), BPEBs **8**, **14** and **20** have relatively higher dielectric anisotropy values, possibly due to the stronger electronegativity of the OCF_3_ group relative to the fluorine atom.

### 2.6. Applications of BPEBs as Blue Phase Liquid Crystal Composition

**Figure 4.**
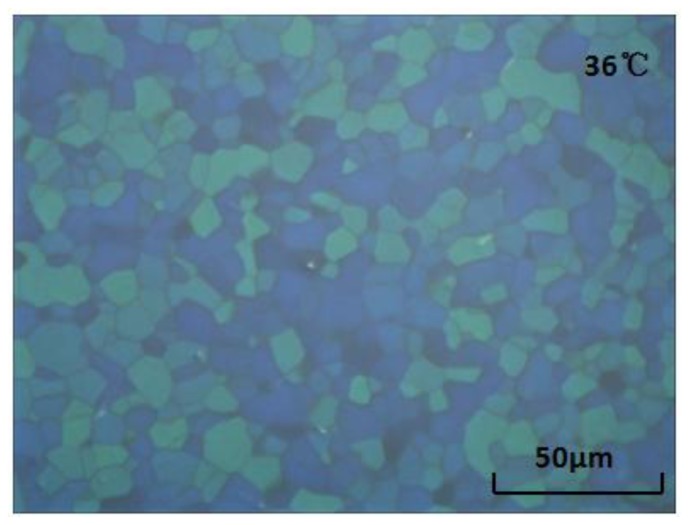
The typical blue phase texture.

Because the synthesized some of BPEBs have high Δ*n* and acceptable Δɛ as described above, we are interested in investigation of the application of them as the compositions in blue phase liquid crystals (BPLC) to possibly increase the Kerr constant, which is key parameter for practical BPLC. After detailed screening the composition and contents, we got a LC mixture containing BPEBs **1**, **3**–**4**, **8**–**9**, **14**, **15**, **21** (5 wt % each) and other liquid crystal mixture (cp: 83.0 °C; Δ*n* = 0.230, and Δɛ = 29.6, at 25 °C), which shows the properties of Δ*n* = 0.283, and Δɛ = 29.0 at 25 °C, which is expected to have high potential applications as BPLC [24]. After adding chiral dopants (R811: 10 wt % and BDH1281: 7 wt %), we obtained a BPLC with a blue phase temperature range of 8 K (from 41 to 33 °C, on the second cooling run). Figure 4 shows a typical BP texture of the obtained BPLC at 36 °C.

## 3. Experimental Section

### 3.1. General Method

All organic starting materials and catalysts are analytically pure and used without further purification. Nuclear magnetic resonance (NMR) spectra were recorded on a JEOL ECA-300 spectrometer (Tokyo, Japan) using CDCl_3_ as solvent at 298 K. ^1^H-NMR (300 MHz) chemical shifts (δ) were referenced to internal standard TMS (for 1H, δ = 0.00 ppm). ^13^C-NMR (75 MHz) chemical shifts were referenced to internal solvent CDCl_3_ (for ^13^C, δ = 77.16 ppm). Mass spectra (MS) were obtained on a Shimadzu GCMS-QP2010S (Kyoto, Japan). Element analyses were obtained with a Flash EA 1112 Element Analyzer (Thermo Fisher Scientific, Waltham, MA, USA). Polarizing microscope LWT300LPT (CEWEI photoelectric technology Co. Ltd., Shanghai, China) equipped with a Weitu WT-3000 hot stage and a TCA 5.0 MP camera was used to observe and record the optical textures of liquid crystal samples. The calorimetric studies were performed on a TA Instruments DSC 2010 (TA Instruments, New Castle, DE, USA) with a heating temperature rate of 10 °C/min. A NAR-4T Abbe refractometer (ATAGO Co. Ltd., Tokyo, Japan) was used to measure optical anisotropy (Δ*n*), and a 3522-50 LCR Hitester (HIOKI E.E. Co., Ueda, Japan) for dielectric anisotropy (Δɛ). All the samples for measuring the Δ*n* and Δɛ were composed of BPEB and nematic host LC at a ratio of 5–10/95–90 (wt %/wt %). The nematic host LC (SLC960524) was prepared by our laboratory, which has the values of Δ*n* = 0.1202 (589 nm) and Δɛ = 3.121 (1000 Hz) at 25 °C.

### 3.2. A Typical Experimental Procedure for Synthesis of BPEB 12 and the Characterization Data of All the BPEBs

As shown in Scheme 2, BPEBs and analogues (**1**–**25**) were synthesized by the similar synthetic route, and their structures were characterized by ^1^H-NMR, ^13^C-NMR (for BPEBs **6** and **7**, the ^13^C-NMR could not be obtained due to their very low solubility in CDCl_3_, DMSO-*d*_6_ or DMF-*d*_7_), and elemental analyses. In this section, the synthetic procedure of BPEB **12** was only described in details, and the characterization data of all the other BPEBs are given.

Preparation of 2,6-difluoro-4-*n*-propylphenyl acetylene (**12c**) (See Scheme S1).

2-Methyl-3-butyn-2-ol (21.8 g, 0.3 mol) was added to a mixture of 2,6-difluoro-4-*n*-propyl-1- iodobenzene (56.4 g, 0.2 mol), tetrakis(triphenylphosphine) palladium(0) (1.0 g, 0.87 mmol), CuBr (0.5 g) and LiBr (2.0 g) in triethylamine (50 mL) with stirring at room temperature. After the mixture was heated at 60 °C for 5 h, it was then cooled to room temperature and the saturated NH_4_Cl aqueous solution (100 mL) and ethyl acetate (200 mL) were added. After separation of the organic phase, the aqueous phase was extracted by ethyl acetate (3 × 150 mL), and the combined organic extracts are dried by K_2_CO_3_ and concentrated on a rotary evaporator, the intermediate **12b** was obtained by column chromatographic separation (40.5 g, 0.17 mol, 85.0%) for the next reaction.

Sodium hydroxide (20.0 g, 0.5 mol) was added to a solution of **12b** (40.0 g) in toluene (150 mL), and then the obtained mixture was heated under reflux for 5 h. After removal of the insoluble excess of Sodium hydroxide by filtration and the solvent under reduced pressure, **12c** was isolated by column chromatography as light yellow oil (17.2 g, 0.095 mol, 55.9%). Characterization data for **12c**: ^1^H-NMR (300 MHz, CDCl_3_) δ 6.70 (d, 2H, ^3^*J*_F–C–C–H_ = 8.4 Hz), 6.67 (s, 1H), 3.44 (s, 1H), 2.52 (t, 2H, *J* = 7.5 Hz), 1.61–1.54 (m, 2H), 0.89 (t, 3H, *J* = 7.5 Hz); ^13^C-NMR (75 MHz, CDCl_3_) δ 163.5 (dd, ^1^*J*_C–F_ = 253.6 Hz, ^5^*J*_C–F_ = 6.0 Hz), 147.1 (t, ^3^*J*_C–F_ = 9.0 Hz), 111.1 (dd, ^2^*J*_C–F_ = 17.1 Hz, ^4^*J*_C–F_ = 6.0 Hz), 98.2 (t, ^2^*J*_C–F_ = 19.8 Hz), 86.5, 70.9, 37.8, 23.7, 13.4; MS *m*/*z* (% rel. intensity) 180 (M^+^, 44), 151 (100); Anal. calcd for C_11_H_10_F_2_: C, 73.33; H, 5.56. Found: C, 73.62; H, 5.59.

Preparation of [2,6-difluoro-4-(2′,6′-difluoro-4′-*n*-propylphenyl)ethynyl]phenyl acetylene (**12g**) (See Scheme S2).

A mixture of 2-bromo-1,3-difluoro-5-iodobenzene (11.16 g, 0.035 mol), ethynyl trimethylsilane (3.8 g, 0.039 mol), tetrakis(triphenylphosphine) palladium(0) (0.5 g, 0.44 mmol) and CuI (1.0 g) in triethylamine (40.0 mL) was stirred in under argon at room temperature for 16 h. After work-up as described for **12b**, **12e** was obtained in 85.7% (10.0 g, 0.03 mol).

A mixture of **12e** (5.6 g), **12c** (3.0 g, 0.017 mol), tetrakis(triphenylphosphine)palladium(0) (0.2 g, 0.17 mmol) and CuI (0.4 g) in triethylamine (40.0 mL) was heated with strring at 60 °C for 5 h, After work-up as described for **12b**, the intermediated **12f** was obtained as colorless solid in 76.4% (4.8 g, 0.013 mol). And then stirring a mixture of **12f** (4.8 g, 0.013 mol) and K_2_CO_3_ (0.1 g, 0.7 mmol) in methanol (150 mL) at room temperature for 5 h, after work up as described for the isolation of **12c**, **12g** was isolated as colorless solid in 84.6% (3.47 g, 0.011 mol). Characterization data for **12g**: ^1^H-NMR (300 MHz, CDCl_3_) δ 7.11 (d, 2H, ^3^*J*_F–C–C–H_ = 7.5 Hz), 6.77 (d, 2H, ^3^*J*_F–C–C–H_ = 8.1 Hz), 3.59 (s, 1H), 2.59 (t, 2H, *J* = 7.5 Hz), 1.68–1.60 (m, 2H), 0.94 (t, 3H, *J* = 7.4 Hz); ^13^C-NMR (300 MHz, CDCl_3_) δ 163.3 (dd, ^1^*J*_C–F_ = 255.0 Hz, ^5^*J*_C–F_ = 6.6 Hz), 162.8 (dd, ^1^*J*_C–F_ = 254.2 Hz, ^5^*J*_C–F_ = 5.8 Hz), 147.6 (t, ^3^*J*_C–F_ = 9.1 Hz), 125.1 (t, ^3^*J*_C–F_ = 11.9 Hz), 114.5 (dd, ^2^*J*_C–F_ = 18.2 Hz, ^4^*J*_C–F_ = 6.8.Hz), 111.4 (dd, ^2^*J*_C–F_ = 17.3 Hz, ^4^*J*_C–F_ = 6.3.Hz), 102.1 (t, ^2^*J*_C–F_ = 19.6 Hz), 98.4 (t, ^2^*J*_C–F_ = 19.6 Hz), 88.8, 88.9, 80.7, 70.5, 38.0, 23.8, 13.6; MS *m*/*z* (% rel. intensity) 316 (M^+^, 56); 287 (100); Anal. calcd for C_19_H_12_F_4_: C, 72.15; H, 3.80. Found: C, 72.52; H, 3.98.

Preparation of 1-[(2′,6′-difluoro-4′-*n*-propylphenyl)ethynyl]-4-[(3″,5″-difluoro-4″-trifluoro-methoxy-phenyl) ethynyl]-3,5-difluorobenzene (**BPEB 12**) (See Scheme S3).

A solution of 4-bromo-2,6-difluoro(trifluoromethoxy)benzene (2.2 g, 8.04 mmol) in toluene (10.0 mL) was added into a solution of **12g** (1.69 g, 5.36 mmol), tetrakis(triphenylphosphine)palladium(0) (0.1 g, 0.09 mmol), and CuI (0.2 g) in triethylamine (20.0 mL) under argon at 60 °C, and then the obtained mixture was stirred at 60 °C for 5 h. After work-up as described for **12c**, the desired **BPEB 12** was isolated as colorless solid in 79.1% (2.17 g, 4.24 mmol). Characterization data for **BPEB 12**: ^1^H-NMR (300 MHz, CDCl_3_) δ 7.24 (d, 2H, ^3^*J*_F–C–C–H_ = 7.5), 7.15 (d, 2H, ^3^*J*_F–C–C–H_ = 7.5 Hz), 6.78 (d, 2H, ^3^*J*_F–C–C–H_ = 8.4 Hz), 2.60 (t, 2H, *J* = 7.5 Hz), 1.69–1.61 (m, 2H), 0.95 (t, 3H, *J* = 7.4 Hz); ^13^C-NMR (75 MHz, CDCl_3_) δ 162.9 (dd, ^1^*J*_C–F_ = 254.3 Hz, ^5^*J*_C–F_ = 5.7 Hz), 162.6 (dd, ^1^*J*_C–F_ = 254.8 Hz, ^5^*J*_C–F_ = 6.0 Hz), 155.9 (dd, ^1^*J*_C–F_ = 253.9 Hz, ^5^*J*_C–F_ = 3.6 Hz ), 147.7 (t, ^3^*J*_C–F_ = 9.0 Hz), 126.3 (t, ^3^*J*_C–F_ = 15.6 Hz), 125.6 (t, ^3^*J*_C–F_ = 12.1 Hz), 123.0 (t, ^3^*J*_C–F_ = 10.6 Hz), 120.5 (q, ^1^*J*_C–F_ = 260.3 Hz), 116.0 (dd, ^2^*J*_C–F_ = 16.2 Hz, ^4^*J*_C–F_ = 5.0 Hz), 114.6 (dd, ^2^*J*_C–F_ = 18.2 Hz, ^4^*J*_C–F_ = 7.0 Hz), 111.4 (dd, ^2^*J*_C–F_ = 17.2 Hz, ^4^*J*_C–F_ = 5.7 Hz), 102.0 (t, ^2^*J*_C–F_ = 18.7 Hz), 98.5 (t, ^2^*J*_C–F_ = 19.7 Hz), 96.8, 95.5, 81.1, 79.2, 38.0, 23.8, 13.6; Anal. calcd for C_26_H_13_F_9_O: C, 60.93; H, 2.54. Found: C, 61.33; H, 2.50.

Characterization data for **BPEB 1**: ^1^H-NMR (300 MHz, CDCl_3_) δ 7.56–7.45 (m, 6H), 6.87 (d, 2H, *J* = 8.8 Hz), 6.78 (d, 2H, *J* = 8.2 Hz), 4.04 (q, 2H, *J* = 7.0 Hz), 2.65 (q, 2H, *J* = 7.6 Hz), 1.42 (t, 3H, *J* = 7.0 Hz), 1.24 (t, 3H, *J* = 7.6 Hz); ^13^C-NMR (75 MHz, CDCl_3_) δ 162.8 (dd, *J* = 253.1 Hz, *J* = 6.0 Hz), 159.3, 147.9 (t, *J* = 8.9 Hz), 133.2, 131.7, 131.4, 124.1, 122.2, 115.0, 114.7, 110.7 (dd, *J* = 16.9 Hz, *J* = 6.3 Hz), 99.4 (t, *J* = 19.9 Hz), 98.1, 91.8, 87.9, 78.9, 63.6, 28.9, 14.9, 14.8; Anal. calcd for C_26_H_20_F_2_O: C, 80.83; H, 5.18. Found: C, 81.03; H, 4.90.

Characterization data for **BPEB 2**: ^1^H-NMR (300 MHz, CDCl_3_) δ 7.56–7.45 (m, 6H), 6.87 (d, 2H, *J* = 8.7 Hz), 6.76 (d, 2H, *J* = 8.0 Hz), 4.04 (q, 2H, *J* = 7.0 Hz), 2.58 (t, 2H, *J* = 7.4 Hz), 1.70–1.58 (m, 2H), 1.42 (t, 3H, *J* = 7.0 Hz), 0.95 (t, 3H, *J* = 7.3 Hz); ^13^C-NMR (75 MHz, CDCl_3_) δ 162.8 (dd, *J* = 253.0 Hz, *J* = 6.0 Hz), 159.3, 146.4 (t, *J* = 9.0 Hz), 133.2, 131.7, 131.4, 124.1, 122.2, 115.0, 114.7, 111.3 (dd, *J* = 16.6 Hz, *J* = 6.0 Hz), 99.4 (t, *J* = 20.1 Hz), 98.1, 91.8, 87.9, 78.3, 63.6, 38.0, 23.9, 14.8, 13.7; Anal. calcd for C_27_H_22_F_2_O: C, 81.00; H, 5.50. Found: C, 81.43; H, 5.74.

Characterization data for **BPEB 3**: ^1^H-NMR (300 MHz, CDCl_3_) δ 7.56–7.45 (m, 6H), 6.87 (d, 2H, *J* = 8.7 Hz), 6.76 (d, 2H, *J* = 8.1 Hz), 4.04 (q, 2H, *J* = 7.0 Hz), 2.60 (t, 2H, *J* = 7.5 Hz), 1.61–1.54 (m, 2H), 1.45–1.30 (m, 5H), 0.94 (t, 3H, *J* = 7.3 Hz); ^13^C-NMR (75 MHz, CDCl_3_) δ 162.8 (dd, *J* = 253.0 Hz, *J* = 6.1 Hz), 159.3, 146.7 (t, *J* = 9.0 Hz), 133.2, 131.7, 131.4, 124.1, 122.2, 115.0, 114.7, 111.2 (dd, *J* = 16.8 Hz, *J* = 6.0 Hz), 99.4 (t, *J* = 20.0 Hz), 98.1, 91.8, 87.9, 78.3, 63.6, 35.7, 32.8, 22.2, 14.8, 13.9; Anal. calcd for C_28_H_24_F_2_O: C, 81.16; H, 5.80. Found: C, 81.53; H, 5.84.

Characterization data for **BPEB 4**: ^1^H-NMR (300 MHz, CDCl_3_) δ 7.55–7.45 (m, 6H), 6.87 (d, 2H, *J* = 8.7 Hz), 6.76 (d, 2H, *J* = 8.1 Hz), 4.04 (q, 2H, *J* = 7.0 Hz), 2.60 (t, 2H, *J* = 7.5 Hz), 1.66–1.57 (m, 2H), 1.43 (t, 3H, *J* = 7.0 Hz), 1.36–1.25 (m, 4H), 0.91 (t, 3H, *J* = 6.9 Hz); ^13^C-NMR (75 MHz, CDCl_3_) δ 162.8 (dd, *J* = 253.2 Hz, *J* = 6.1 Hz), 159.3, 146.7 (t, *J* = 9.0 Hz), 133.2, 131.7, 131.4, 124.1, 122.2, 115.0, 114.6, 111.2 (dd, *J* = 17.0 Hz, *J* = 6.1 Hz), 99.4 (t, *J* = 19.5 Hz), 98.1, 91.8, 87.9, 78.3, 63.6, 35.9, 31.4, 30.4, 22.5, 14.8, 14.1; Anal. calcd for C_29_H_26_F_2_O: C, 81.31; H, 6.07. Found: C, 81.26; H, 6.34.

Characterization data for **BPEB 5**: ^1^H-NMR (300 MHz, CDCl_3_) δ 7.54–7.39 (m, 7H), 6.94 (d, 2H, *J* = 8.4 Hz), 6.87 (d, 2H, *J* = 8.3 Hz), 4.04 (q, 2H, *J* = 6.7 Hz), 2.60 (t, 3H, *J* = 7.3 Hz), 1.72–1.59 (m, 2H), 1.43 (t, 3H, *J* = 6.8 Hz), 0.96 (t, 3H, *J* = 7.2 Hz); ^13^C-NMR (75 MHz, CDCl_3_) δ 162.7 (d, *J* = 251.6 Hz), 159.3, 146.1 (d, *J* = 7.2 Hz), 133.2, 133.1, 131.6, 131.4, 124.3, 123.8, 122.6, 115.5 (d, *J* = 20.3 Hz), 114.9 (d, *J* = 27.2 Hz), 114.7, 108.9 (d, *J* = 16.0 Hz), 93.6, 91.6, 88.0, 84.8, 63.6, 37.9, 24.1, 14.9, 13.8; Anal. calcd for C_27_H_23_FO: C, 84.82; H, 6.02. Found: C, 84.88; H, 6.13.

Characterization data for **BPEB 6**: ^1^H-NMR (300 MHz, CDCl_3_) δ 7.64–7.53 (m, 8H), 7.49–7.45 (m, 2H), 7.41 (d, 1H, *J* = 7.5 Hz), 6.96–6.92 (m, 2H), 6.88 (d, 2H, *J* = 8.7 Hz), 4.06 (q, 2H, *J* = 7.0 Hz), 2.61 (t, 2H, *J* = 7.3 Hz), 1.72–1.60 (m, 2H), 1.43 (t, 3H, *J* = 7.0 Hz), 0.95 (t, 3H, *J* = 7.3 Hz); Anal. calcd for C_33_H_27_FO: C, 86.46; H, 5.90. Found: C, 86.98; H, 5.93.

Characterization data for **BPEB 7**: ^1^H-NMR (300 MHz, CDCl_3_) δ 7.66–7.55 (m, 8H), 7.48 (d, 2H, *J* = 8.8 Hz), 6.88 (d, 2H, *J* = 8.8 Hz), 6.77 (d, 2H, *J* = 8.1 Hz), 4.06 (q, 2H, *J* = 7.0 Hz), 2.59 (t, 2H, *J* = 7.4 Hz), 1.71–1.59 (m, 2H), 1.43 (t, 3H, *J* = 7.0 Hz), 0.95 (t, 3H, *J* = 7.4 Hz); Anal. calcd for C_33_H_26_F_2_O: C, 83.19; H, 5.46. Found: C, 83.26; H, 5.72.

Characterization data for **BPEB 8**: ^1^H-NMR (300 MHz, CDCl_3_) δ 7.58–7.48 (m, 4H), 7.17 (d, 2H, *J* = 7.8 Hz), 6.77 (d, 2H, *J* = 8.1 Hz), 2.59 (t, 2H, *J* = 7.4 Hz),1.71–1.59 (m, 2H), 0.95 (t, 3H, *J* = 7.3 Hz); ^13^C-NMR (75 MHz, CDCl_3_) δ 162.8 (dd, *J* = 253.3 Hz, *J* = 5.8 Hz), 155.8 (dd, *J* = 255.8 Hz, *J* = 3.6 Hz), 146.8 (t, *J* =8.9 Hz), 131.8, 131.7, 125.6 (t, *J* = 17.2 Hz), 123.8 (t, *J* = 9.1 Hz), 122.1, 120.6 (q, *J* = 259.6 Hz), 115.8 (d, *J* = 17.5 Hz, *J* = 6.2 Hz), 111.3 (dd, *J* = 16.9 Hz, *J* = 6.1 Hz), 99.2 (t, *J* = 19.9 Hz), 97.6, 92.0, 87.9, 79.1, 38.0, 23.8, 13.6; Anal. calcd for C_26_H_15_F_7_O: C, 65.55; H, 3.15. Found: C, 64.98; H, 3.13.

Characterization data for **BPEB 9**: ^1^H-NMR (300 MHz, CDCl_3_) δ 7.57–7.47 (m, 4H), 7.16–7.11 (m, 2H), 6.76 (d, 2H, *J* = 8.1 Hz), 2.59 (t, 2H, *J* = 7.4 Hz), 1.71–1.58 (m, 2H), 0.95 (t, 3H, *J* = 7.3 Hz); ^13^C-NMR (75 MHz, CDCl_3_) δ 162.8 (dd, *J* = 253.1 Hz, *J* = 5.8 Hz), 151.1 (ddd, *J* = 251.1 Hz, *J* = 10.4 Hz, *J* = 4.9 Hz), 146.7 (t, *J* = 9.0 Hz), 140.5 (dt, *J* = 255.7 Hz, *J* = 15.3 Hz), 131.8, 131.7, 123.5, 122.4, 119.0 9 (td), 116.0 (ddd, *J* = 15.2 Hz, *J* = 7.3 Hz), 111.3 (dd, *J* =17.0 Hz, *J* = 5.9 Hz), 99.2 (t, *J* = 20.0 Hz), 97.7, 90.6, 88.2, 78.9, 38.0, 23.9, 13.6; Anal. calcd for C_25_H_15_F_5_: C, 73.17; H, 3.66. Found: C, 73.02; H, 3.51.

Characterization data for **BPEB 10**: ^1^H-NMR (300 MHz, CDCl_3_) δ 7.47 (t, 1H, J = 7.6 Hz), 7.35–7.30 (m, 2H), 7.20 (d, 2H, *J* = 7.8 Hz), 6.77 (d, 2H, *J* = 8.2 Hz), 2.59 (t, 2H, *J* = 7.4 Hz), 1.71–1.59 (m, 2H), 0.95 (t, 3H, *J* = 7.3 Hz); ^13^C-NMR (75 MHz, CDCl_3_) δ 162.8 (dd, *J* = 253.9 Hz, *J* = 6.0 Hz), 162.3 (d, *J* = 253.5 Hz), 155.8 (dd, *J* = 255.4 Hz, *J* = 3.8 Hz), 147.2 (t, *J* = 9.1 Hz), 133.3, 127.6 (d, *J* = 3.2 Hz), 126.1 (t, *J* = 15.8 Hz), 125.6 (d, *J* = 9.3 Hz), 123.3 (t, *J* = 11.0 Hz), 120.5 (q, *J* = 261.0 Hz), 118.7 (d, *J* = 22.7 Hz), 115.9 (dd, *J* = 15.5 Hz, *J* = 4.3 Hz), 111.4 (dd, *J* = 17.5 Hz, *J* = 5.6 Hz), 111.1, 98.8 (t, *J* = 19.6 Hz), 96.4, 92.6, 85.5, 80.0, 40.0, 23.8, 13.6; Anal. calcd for C_26_H_14_F_8_O: C, 63.16; H, 2.83. Found: C, 63.03; H, 2.56.

Characterization data for **BPEB 11**: ^1^H-NMR (300 MHz, CDCl_3_) δ 7.45 (t, 1H, *J* = 7.4 Hz), 7.36–7.26 (m, 2H), 7.21–7.14 (m, 2H), 6.77 (d, 2H, *J* = 8.2 Hz), 2.59 (t, 2H, *J* = 7.3 Hz), 1.71–1.58 (m, 2H), 0.95 (t, 3H, *J* = 7.3 Hz); ^13^C-NMR (75 MHz, CDCl_3_) δ 162.8 (dd, *J* = 253.6 Hz, *J* = 5.6 Hz), 162.3 (d, *J* = 253.2 Hz), 151.1 (ddd, *J* = 251.0 Hz, *J* = 10.5 Hz, *J* = 4.8 Hz), 147.2 (t, *J* = 8.9 Hz), 140.6 (dt, *J* = 250.7 Hz, *J* = 10.9 Hz), 133.3, 127.6 (d, *J* = 3.3 Hz), 125.3 (d, *J* = 9.3 Hz), 118.6 (dd, *J* = 12.4 Hz, *J* = 6.9 Hz), 116.1 (dd, *J* = 15.5 Hz, *J* = 7.4 Hz), 111.4, 111.4 (dd, *J* = 17.8 Hz, *J* = 6.3 Hz), 98.9 (t, *J* = 19.4 Hz), 96.5 (d, *J* = 3.2 Hz), 92.9, 84.1, 79.9, 38.0, 23.8, 13.6; Anal. calcd for C_25_H_14_F_6_: C, 70.09; H, 3.27. Found: C, 70.33; H, 3.46.

Characterization data for **BPEB 13**: ^1^H-NMR (300 MHz, CDCl_3_) δ 7.22–7.12 (m, 4H), 6.78 (d, 2H, *J* = 8.2 Hz), 2.60 (t, 2H, *J* = 7.4 Hz), 1.71–1.59 (m, 2H), 0.95 (t, 3H, *J* = 7.3 Hz); _13_C-NMR (75 MHz, CDCl_3_) δ 162.8 (dd, *J* = 254.0 Hz, *J* = 5.4 Hz), 162.6 (dd, *J* = 255.1 Hz, *J* = 6.4 Hz), 151.2 (ddd, *J* = 252.3 Hz, *J* = 10.7 Hz, *J* = 4.4 Hz), 147.6 (t, *J* = 6.4 Hz), 140.9 (dt, *J* = 256.0 Hz, *J* = 15.4 Hz), 125.3 (t, *J* = 12.2 Hz), 118.4, 116.2 (dd, *J* = 15.5 Hz, *J* = 7.6 Hz), 114.5 (dd, *J* =17.7 Hz, *J* = 7.1 Hz), 111.4 (dd, *J* = 16.4 Hz, *J* = 5.1 Hz), 102.3 (t, *J* = 21.5 Hz), 98.6 (t, *J* = 20.2 Hz), 97.3, 95.6, 80.9, 77.8, 38.0, 23.7, 13.5; Anal. calcd for C_25_H_13_F_7_: C, 67.26; H, 2.91. Found: C, 69.52; H, 2.66.

Characterization data for **BPEB 14**: ^1^H-NMR (300 MHz, CDCl_3_) δ 7.57–7.48 (m, 4H), 7.17 (d, 2H, *J* = 7.9 Hz), 6.77 (d, 2H, *J* = 8.1 Hz), 2.61 (t, 2H, *J* = 7.5 Hz), 1.65–1.55 (m, 2H), 1.42–1.30 (m, 2H), 0.94 (t, 3H, *J* = 7.3 Hz); ^13^C-NMR (75 MHz, CDCl_3_) δ 162.8 (dd, *J* = 253.4 Hz, *J* = 6.2 Hz), 155.8 (dd, *J* = 255.3 Hz, *J* = 3.7 Hz), 147.0 (t, *J* = 9.1 Hz), 131.8, 131.7, 125.8 (t, *J* = 16.3 Hz), 123.8 (t, *J* = 8.8 Hz), 122.1, 120.4 (q, *J* = 258.9 Hz), 115.8 (dd, *J* = 17.5 Hz, *J* = 6.2 Hz), 111.3 (dd, *J* = 16.7 Hz, *J* = 5.7 Hz), 99.2 (t, *J* = 20.1 Hz), 97.6, 92.0, 87.9, 79.0, 35.7, 32.8, 22.3, 13.9; Anal. calcd for C_27_H_17_F_7_O: C, 66.12; H, 3.47. Found: C, 66.01; H, 3.36.

Characterization data for **BPEB 15**: ^1^H-NMR (300 MHz, CDCl_3_) δ 7.56–7.46 (m, 4H), 7.13 (t, 2H, *J* = 6.9 Hz), 6.76 (d, 2H, *J* = 8.1 Hz), 2.60 (t, 2H, *J* = 7.4 Hz), 1.64–1.54 (m, 2H), 1.42–1.30 (m, 2H), 0.94 (t, 3H, *J* = 7.3 Hz); ^13^C-NMR (75 MHz, CDCl_3_) δ 162.8 (dd, *J* = 253.4 Hz, *J* = 6.0 Hz), 151.1 (ddd, *J* = 250.6 Hz, *J* = 10.1 Hz, *J* = 4.2 Hz), 146.9 (t, *J* = 9.0 Hz), 140.4 (dt, *J* = 254.7 Hz, *J* = 14.9 Hz), 131.8, 131.7, 123.5, 122.4, 119.1 (td), 116.0 (ddd, *J* = 15.4 Hz, *J* = 7.3 Hz, *J* = 4.8 Hz), 111.2 (dd, *J* =17.1 Hz, *J* = 5.9 Hz), 99.2 (t, *J* = 19.8 Hz), 97.7, 90.6, 88.2, 78.9, 35.7, 32.8, 22.3, 13.9; Anal. calcd for C_26_H_17_F_5_: C, 73.58; H, 4.01. Found: C, 73.51; H, 3.96.

Characterization data for **BPEB 16**: ^1^H-NMR (300 MHz, CDCl_3_) δ 7.47 (t, 1H, *J* = 7.5 Hz), 7.39–7.29 (m, 2H), 7.20 (d, 2H, *J* = 7.7 Hz), 6.77 (d, 2H, *J* = 8.2 Hz), 2.61 (t, 2H, *J* = 7.6 Hz), 1.65–1.55 (m, 2H), 1.42–1.30 (m, 2H), 0.94 (t, 3H, *J* = 7.3 Hz); ^13^C-NMR (75 MHz, CDCl_3_) δ 162.8 (dd, *J* = 253.8 Hz, *J* = 5.8 Hz), 162.3 (d, *J* = 253.7 Hz), 155.8 (dd, *J* = 255.5 Hz, *J* = 3.7 Hz), 147.5 (t, *J* = 8.9 Hz), 133.3, 127.6 (d, *J* = 3.4 Hz), 126.1 (t, *J* = 15.8 Hz), 125.6 (d, *J* = 9.4 Hz), 123.3 (t, *J* = 11.2 Hz), 120.5 (q, *J* = 261.0 Hz), 118.7 (d, *J* = 22.8 Hz), 115.9 (dd, *J* = 16.2 Hz, *J* = 6.4 Hz), 111.3 (dd, *J* = 16.1 Hz, J = 5.8 Hz), 111.1, 98.8 (t, *J* = 19.5 Hz), 96.4, 92.5, 85.5, 80.0, 35.7, 32.8, 22.3, 13.9; Anal. calcd for C_27_H_16_F_8_O: C, 63.78; H, 3.15. Found: C, 63.61; H, 3.26.

Characterization data for **BPEB 17**: ^1^H-NMR (300 MHz, CDCl_3_) δ 7.44 (t, 1H, *J* = 7.4 Hz), 7.33–7.27 (m, 2H), 7.16 (t, 2H, *J* = 6.6 Hz), 6.76 (d, 2H, *J* = 8.2 Hz), 2.60 (t, 2H, *J* = 7.5 Hz), 1.64–1.54 (m, 2H), 1.42–1.29 (m, 2H), 0.94 (t, 3H, *J* = 7.3 Hz); ^13^C-NMR (75 MHz, CDCl_3_) δ 162.8 (dd, *J* = 253.7 Hz, *J* = 5.8 Hz), 162.3 (d, *J* = 253.4 Hz), 151.1 (ddd, *J* = 250.9 Hz, *J* = 10.4 Hz, *J* = 4.3 Hz), 147.4 (t, *J* = 9.0 Hz), 140.7 (dt, *J* = 255.1 Hz, *J* = 14.7 Hz), 133.3, 127.6 (d, *J* = 3.3 Hz), 125.3 (d, *J* = 9.5 Hz), 118.6 (dd, *J* = 14.2 Hz, *J* = 9.1 Hz), 116.1 (dd, *J* = 15.4 Hz, *J* = 7.3 Hz), 111.5, 111.3 (dd, *J* = 17.0 Hz, J = 5.9 Hz), 98.8 (t, *J* = 19.6 Hz), 96.5 (d, *J* = 3.1 Hz), 92.9, 84.1, 79.9, 35.7, 32.8, 22.3, 13.9; Anal. calcd for C_26_H_16_F_6_: C, 70.59; H, 3.62. Found: C, 70.68; H, 3.36.

Characterization data for **BPEB 18**: ^1^H-NMR (300 MHz, CDCl_3_) δ 7.23 (d, 2H, *J* = 7.7 Hz), 7.14 (d, 2H, *J* = 7.3 Hz), 6.78 (d, 2H, *J* = 8.2 Hz), 2.62 (t, 2H, *J* = 7.6 Hz), 1.65–1.55 (m, 2H), 1.41–1.30 (m, 2H), 0.94 (t, 3H, *J* = 7.3 Hz); ^13^C-NMR (75 MHz, CDCl_3_) δ 162.9 (dd, *J* = 254.1 Hz, *J* = 5.8 Hz), 162.7 (dd, *J* = 255.4 Hz, *J* = 6.2 Hz), 155.9 (dd, *J* = 255.8 Hz, *J* = 3.6 Hz), 147.9 (t, *J* = 9.0 Hz), 126.3 (t, *J* = 15.8 Hz), 125.6 (t, *J* = 12.0 Hz), 123.0 (t, *J* = 10.1 Hz), 120.5 (q, *J* = 260.8 Hz), 116.0 (dd, *J* = 17.0 Hz, *J* = 6.6 Hz), 114.6 (dd, *J* = 17.8 Hz, *J* = 7.9 Hz), 111.3 (dd, *J* = 17.0 Hz, *J* = 5.6 Hz), 102.0 (t, *J* = 18.5 Hz), 98.2 (t, *J* = 19.9 Hz), 96.9, 95.5, 81.1, 79.2, 35.7, 32.7, 22.2, 13.8; Anal. calcd for C_27_H_15_F_9_O: C, 61.60; H, 2.85. Found: C, 60.99; H, 2.64.

Characterization data for **BPEB 19**: ^1^H-NMR (300 MHz, CDCl_3_) δ 7.20 (t, 2H, *J* = 6.7 Hz), 7.12 (d, 2H, *J* = 7.1 Hz), 6.77 (d, 2H, *J* = 8.2 Hz), 2.62 (t, 2H, *J* = 7.5 Hz), 1.65–1.55 (m, 2H), 1.42–1.30 (m, 2H), 0.94 (t, 3H, *J* = 7.3 Hz); ^13^C-NMR (75 MHz, CDCl_3_) δ 162.8 (dd, *J* = 254.1 Hz, *J* = 5.7 Hz), 162.6 (dd, *J* = 254.7 Hz, *J* = 6.2 Hz), 151.1 (ddd, *J* = 251.3 Hz, *J* = 10.4 Hz, *J* = 4.4 Hz), 147.9 (t, *J* = 9.2 Hz), 140.9 (dt, *J* = 256.0 Hz, *J* = 15.6 Hz), 125.2 (t, *J* = 12.0 Hz), 118.4 (td), 116.3 (ddd, *J* = 15.4 Hz, *J* = 7.3 Hz), 114.6 (dd, *J* =18.2 Hz, *J* = 7.6 Hz), 111.4 (dd, *J* = 17.2 Hz, *J* = 5.7 Hz), 102.2 (t, *J* = 19.8 Hz), 98.4 (t, *J* = 20.1 Hz), 97.3, 95.6, 80.9, 77.8, 35.7, 32.8, 22.3, 13.9; Anal. calcd for C_26_H_15_F_7_: C, 67.83; H, 3.26. Found: C, 67.77; H, 3.44.

Characterization data for **BPEB 20**: ^1^H-NMR (300 MHz, CDCl_3_) δ 7.57–7.48 (m, 4H), 7.16 (d, 2H, *J* = 7.7 Hz), 6.77 (d, 2H, *J* = 8.2 Hz), 2.60 (t, 2H, *J* = 7.5 Hz), 1.65–1.56 (m, 2H), 1.38–1.28 (m, 4H), 0.91 (t, 3H, *J* = 6.7 Hz); ^13^C-NMR (75 MHz, CDCl_3_) δ 162.8 (dd, *J* = 253.4 Hz, *J* = 6.0 Hz), 155.8 (dd, *J* = 255.4 Hz, *J* = 3.7 Hz), 147.0 (t, *J* = 9.1 Hz), 131.8, 131.7, 125.8 (t, *J* = 16.5 Hz), 123.8 (t, *J* = 8.7 Hz), 122.2, 120.6 (q, *J* = 259.6 Hz), 115.8 (dd, *J* = 17.6 Hz, *J* = 6.4 Hz), 111.2 (dd, *J* = 17.1 Hz, *J* = 6.1 Hz), 99.1 (t, *J* = 19.9 Hz), 97.6, 92.0, 87.9, 79.1, 35.9, 31.3, 30.3, 22.5, 14.0; Anal. calcd for C_28_H_19_F_7_O: C, 66.67; H, 3.77. Found: C, 66.35; H, 3.46.

Characterization data for **BPEB 21**: ^1^H-NMR (300 MHz, CDCl_3_) δ 7.56–7.46 (m, 4H), 7.16–7.11 (m, 2H), 6.76 (d, 2H, *J* = 8.2 Hz), 2.60 (t, 2H, *J* = 7.6 Hz), 1.66–1.56 (m, 2H), 1.38–1.29 (m, 4H), 0.91 (t, 3H, *J* = 6.6 Hz); ^13^C-NMR (75 MHz, CDCl_3_) δ 162.8 (dd, *J* = 253.2 Hz, *J* = 6.0 Hz), 151.1 (ddd, *J* = 250.7 Hz, *J* = 10.3 Hz, *J* = 4.5 Hz), 147.0 (t, *J* = 8.9 Hz), 140.5 (dt, *J* = 256.0 Hz, *J* = 15.6 Hz), 131.8, 131.7, 123.5, 122.4, 119.1 (td), 116.0 (ddd, *J* = 15.2 Hz, *J* = 7.2 Hz, *J* = 4.3 Hz), 111.2 (dd, *J* = 16.6 Hz, *J* = 5.6 Hz), 99.2 (t, *J* = 19.7 Hz), 97.7, 90.6, 88.2, 78.9, 35.9, 31.3, 30.4, 22.5, 14.0; Anal. calcd for C_27_H_19_F_5_: C, 73.97; H, 4.34. Found: C, 73.49; H, 4.44.

Characterization data for **BPEB 22**: ^1^H-NMR (300 MHz, CDCl_3_) δ 7.46 (t, 1H, *J* = 7.5 Hz), 7.34–7.29 (m, 2H), 7.19 (d, 2H, *J* = 7.1 Hz), 6.77 (d, 2H, *J* = 8.2 Hz), 2.60 (t, 2H, *J* = 7.5 Hz), 1.61 (m, 2H), 1.36–1.30 (m, 4H), 0.91 (t, 3H, *J* = 6.6 Hz); ^13^C-NMR (75 MHz, CDCl_3_) δ 162.8 (dd, *J* = 253.9 Hz, *J* = 5.8 Hz), 162.3 (d, *J* = 253.6 Hz), 155.8 (dd, *J* = 255.5 Hz, *J* = 3.5 Hz), 147.5 (t, *J* = 9.0 Hz), 133.3, 127.6 (d, *J* = 3.4 Hz), 126.1 (t, *J* = 15.8 Hz), 125.6 (d, *J* = 9.5 Hz), 123.3 (t, *J* = 10.9 Hz), 120.5 (q, *J* = 261.0 Hz), 118.7 (d, *J* = 22.7 Hz), 115.9 (dd, *J* = 16.0 Hz, *J* = 6.5 Hz), 111.3 (dd, *J* = 14.6 Hz, *J* = 6.1 Hz), 111.1, 98.8 (t, *J* = 19.9 Hz), 96.4, 92.6, 85.5, 80.0, 36.0, 31.3, 30.3, 22.5, 14.0; Anal. calcd for C_28_H_18_F_8_O: C, 64.37; H, 3.45. Found: C, 63.89; H, 3.26.

Characterization data for **BPEB 23**: ^1^H-NMR (300 MHz, CDCl_3_) δ 7.44 (t, 1H, *J* = 7.6 Hz), 7.32–7.27 (m, 2H), 7.16 (d, 2H, *J* = 6.8 Hz), 6.77 (d, 2H, *J* = 8.1 Hz), 2.60 (t, 2H, J = 7.9 Hz), 1.61 (m, 2H), 1.36–1.29 (m, 4H), 0.91 (t, 3H, *J* = 6.6 Hz); ^13^C-NMR (75 MHz, CDCl_3_) δ 162.8 (dd, *J* = 253.8 Hz, *J* = 5.8 Hz), 162.3 (d, *J* = 253.4 Hz), 151.1 (ddd, *J* = 251.1 Hz, *J* = 10.4 Hz, *J* = 4.4 Hz), 147.4 (t, *J* = 9.1 Hz), 140.7 (dt, *J* = 256.0Hz, *J* = 15.5 Hz), 133.2, 127.6 (d, *J* = 3.2 Hz), 125.3 (d, *J* = 9.5 Hz), 118.6 (dd, *J* = 15.6 Hz, *J* = 6.9 Hz), 116.1 (dd, *J* = 15.3 Hz, *J* = 7.3 Hz), 111.5, 111.3 (dd, *J* = 17.1 Hz, *J* = 6.9 Hz), 98.9 (t, *J* = 19.9 Hz), 96.5, 92.9, 84.1, 79.9, 36.0, 31.4, 30.3, 22.5, 14.0; Anal. calcd for C_27_H_18_F_6_: C, 71.05; H, 3.95. Found: C, 71.17; H, 4.11.

Characterization data for **BPEB 24**: ^1^H-NMR (300 MHz, CDCl_3_) δ 7.23 (d, 2H, *J* = 7.7 Hz), 7.14 (d, 2H, *J* = 7.2 Hz), 6.78 (d, 2H, *J* = 8.2 Hz), 2.61 (t, 2H, *J* = 7.5 Hz), 1.61 (m, 2H), 1.38–1.27 (m, 4H), 0.90 (t, 3H, *J* = 6.9 Hz); ^13^C-NMR (75 MHz, CDCl_3_) δ 162.9 (dd, *J* = 254.7 Hz, *J* = 6.4 Hz), 162.6 (dd, *J* = 254.8 Hz, *J* = 6.1 Hz), 155.8 (dd, *J* = 255.7 Hz, *J* = 3.6 Hz), 148.0 (t, *J* = 9.4 Hz), 126.3 (t, *J* = 15.8 Hz), 125.5 (t, *J* = 12.6 Hz), 123.0 (t, *J* = 10.6 Hz), 120.5 (q, *J* = 260.4 Hz), 116.0 (dd, *J* = 17.5 Hz, *J* = 6.2 Hz), 114.6 (dd, *J* = 18.1 Hz, *J* = 7.3 Hz), 111.4 (dd, *J* = 17.2 Hz, *J* = 5.5 Hz), 102.0 (t, *J* = 17.6 Hz), 98.4 (t, *J* = 19.9 Hz), 96.9, 95.5, 81.1, 79.2, 36.0, 31.3, 30.3, 22.5, 14.0; Anal. calcd for C_28_H_17_F_9_O: C, 62.22; H, 3.15. Found: C, 62.18; H, 3.12.

Characterization data for **BPEB 25**: ^1^H-NMR (300 MHz, CDCl_3_) δ 7.19 (t, 2H, *J* = 6.8 Hz), 7.12 (d, 2H, *J* = 7.2 Hz), 6.77 (d, 2H, *J* = 8.2 Hz), 2.61 (t, 2H, *J* = 7.5 Hz), 1.61 (m, 2H), 1.34–1.32 (m, 4H), 0.90 (t, 3H, *J* = 6.6 Hz); ^13^C-NMR (75 MHz, CDCl_3_) δ 162.9 (dd, *J* = 254.1 Hz, *J* = 5.6 Hz), 162.6 (dd, *J* = 255.0 Hz, *J* = 6.4 Hz), 151.2 (ddd, *J* = 251.5 Hz, *J* = 10.6 Hz, *J* = 4.9 Hz), 147.9 (t, *J* = 9.6 Hz), 140.9 (dt, *J* = 256.8 Hz, *J* = 15.5 Hz), 125.2 (t, *J* = 11.6 Hz), 118.4 (td), 116.2 (ddd, *J* = 15.4 Hz, *J* = 7.6 Hz), 114.5 (dd, *J* = 18.5 Hz, *J* = 7.6 Hz), 111.3 (dd, *J* = 17.9 Hz, *J* = 5.2 Hz), 102.2 (t, *J* = 19.6 Hz), 98.5 (t, *J* = 20.1 Hz), 97.2, 95.6, 80.9, 77.8, 36.0, 31.3, 30.3, 22.5, 13.9; Anal. calcd for C_27_H_17_F_7_: C, 68.35; H, 3.59. Found: C, 68.38; H, 3.76.

## 4. Conclusions

In summary, we have designed and synthesized BPEBs by convenient Sonogashira cross-coupling reactions, which have different numbers of side-substitute fluorine atoms on benzene rings, and alkyl chains, ethoxyl groups, fluorine atoms and trifluoromethyl groups as the end groups. The detailed investigation of the synthesized BPEBs properties have disclosed that the melting points, clearing points, nematic phase, optical anisotropy (Δ*n*) and dielectric anisotropy (Δɛ) greatly depend on both the numbers of side-substitute fluorine atoms and structures of the end groups. On the basis of the obtained properties of the synthesized BPEBs, some of them have been expected to have high potential application as the compositions in blue phase liquid crystals. Therefore, a mixture of blue phase liquid crystal has been prepared with a relative wide blue phase temperature range of 8 °C. The obtained results have implied that the synthesized BPEBs will certainly be important in the development of new types and properties of LCs. Further study on the application of BPEBs in making other new types of LCs is underway in our laboratory.

## Supplementary Information



## Figures and Tables

**Table 1.** Melting points and clearing points of 1,4-bis(phenylethynyl)benzene derivatives (BPEBs) and analogues by DSC.

BPEB	Structure	*Mp* (°C)	Δ*H* (J/g)	*Cp* (°C)	Δ*H* (J/g)
**1**	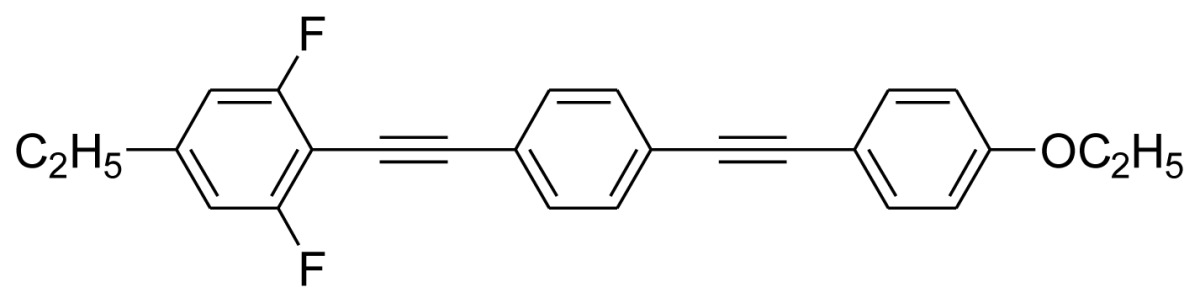	129.5	62.9	251.2	3.0
**2**	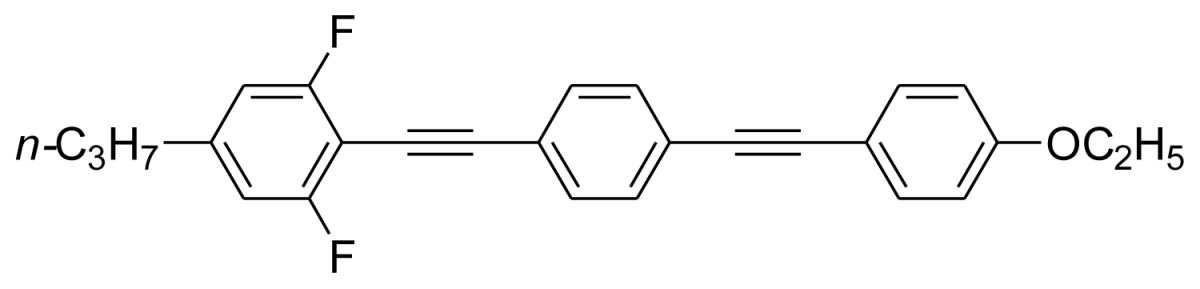	130.2	67.8	254.2	3.8
**3**	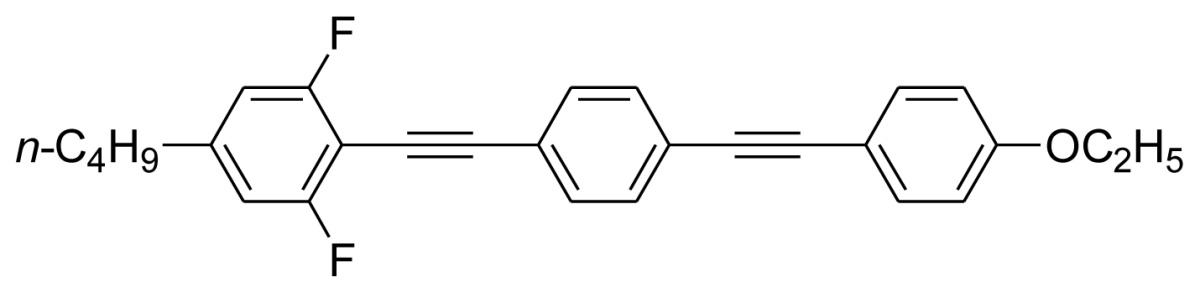	91.0	63.7	238.0	3.8
**4**	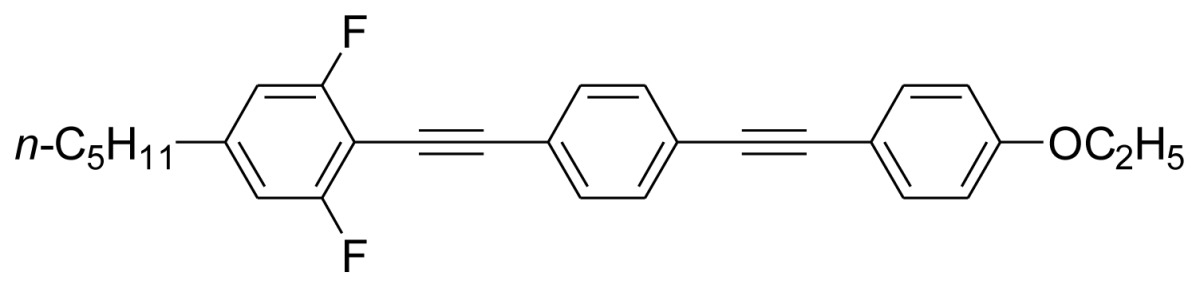	88.7	55.7	232.4	3.8
**5**	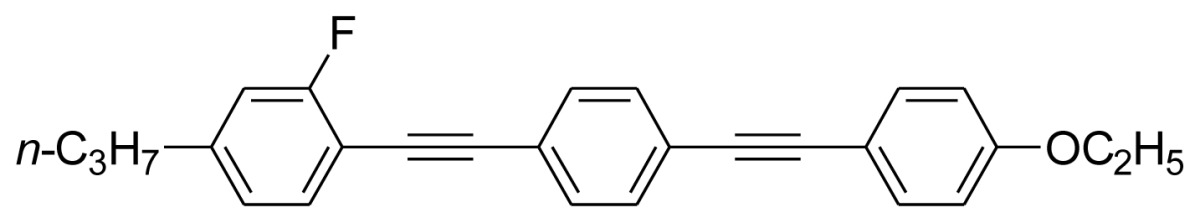	140.4	46.0	262.0	3.0
**6**	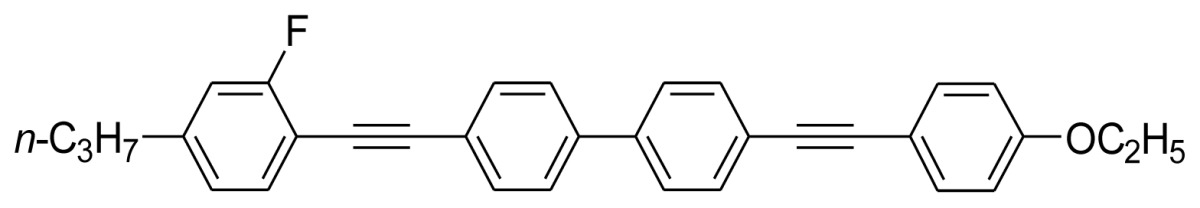	213.2	41.2	–	–
**7**	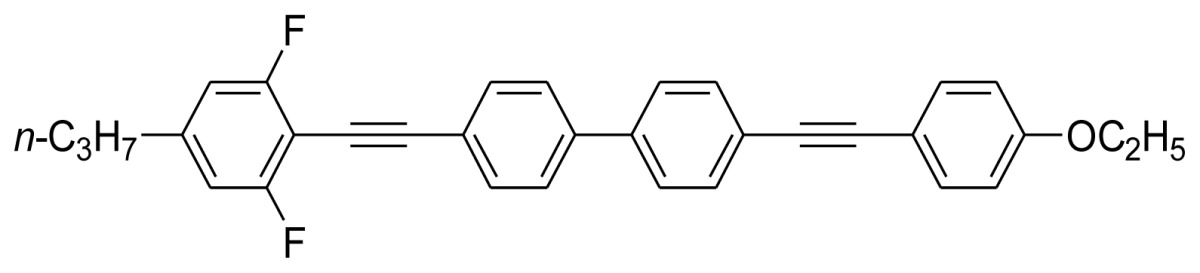	190.4	57.3	–	–
**8**	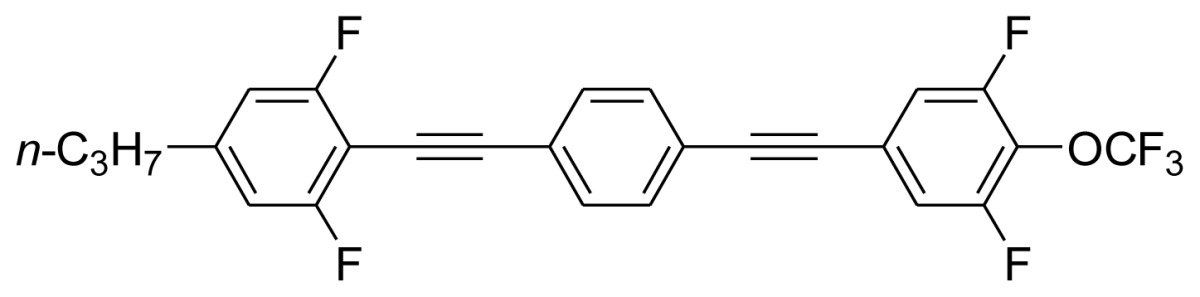	131.1	59.8	152.9	5.8
**9**	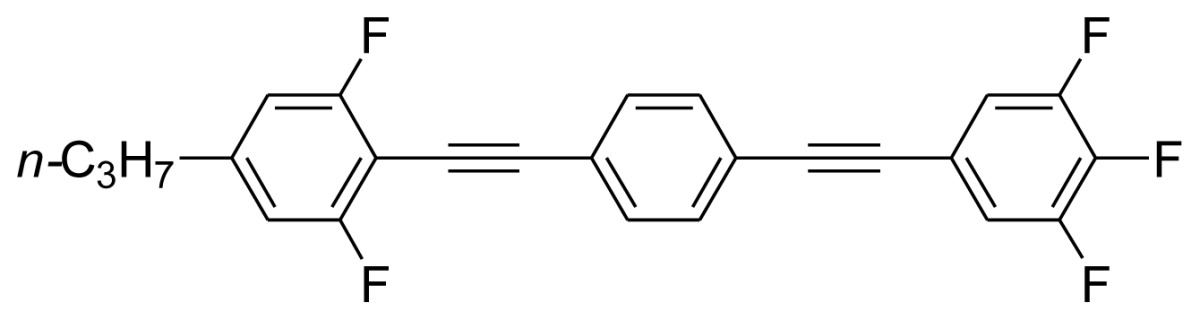	141.4	88.5	a	–
**10**	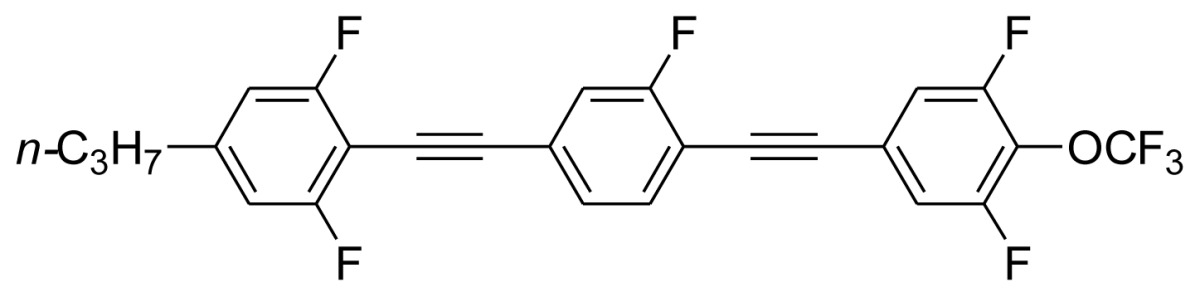	150.7	68.7	–	–
**11**	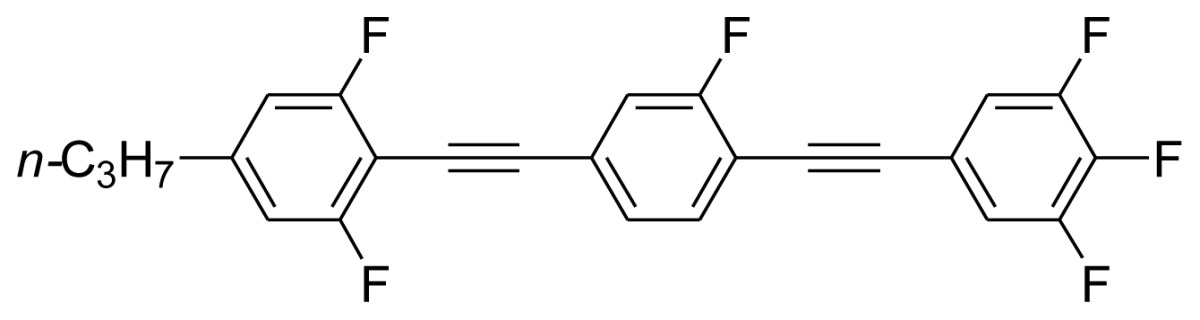	142.8	77.4	–	–
**12**	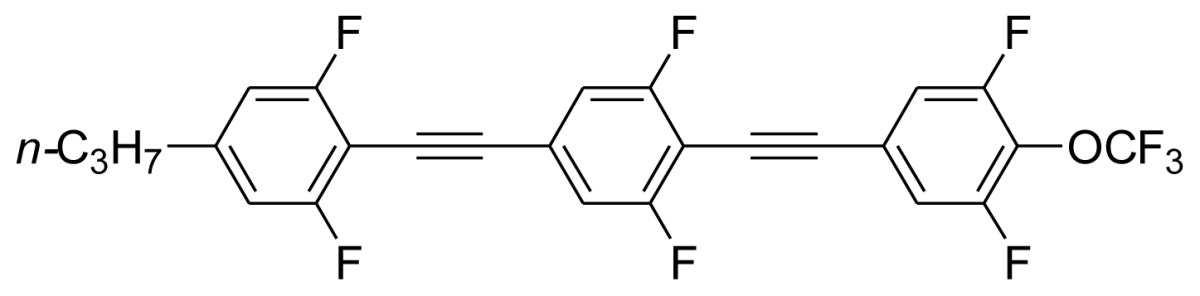	178.2	54.0	–	–
**13**	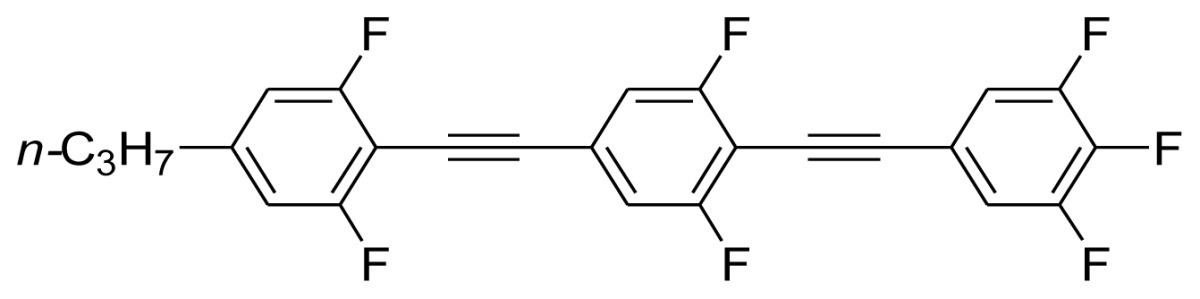	176.4	66.5	–	–
**14**	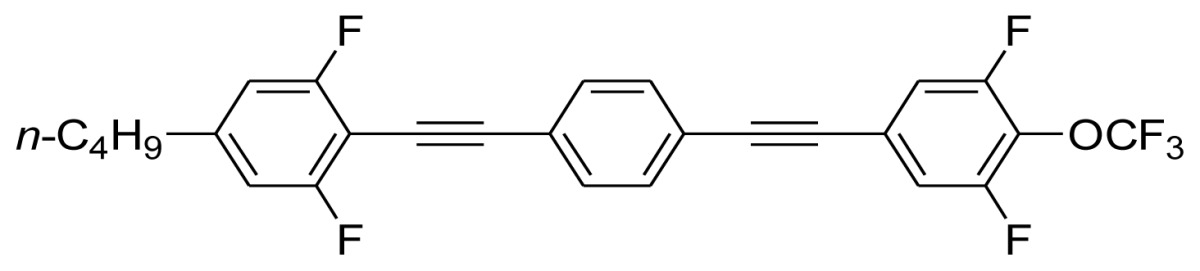	142.6	69.3	b	–
**15**	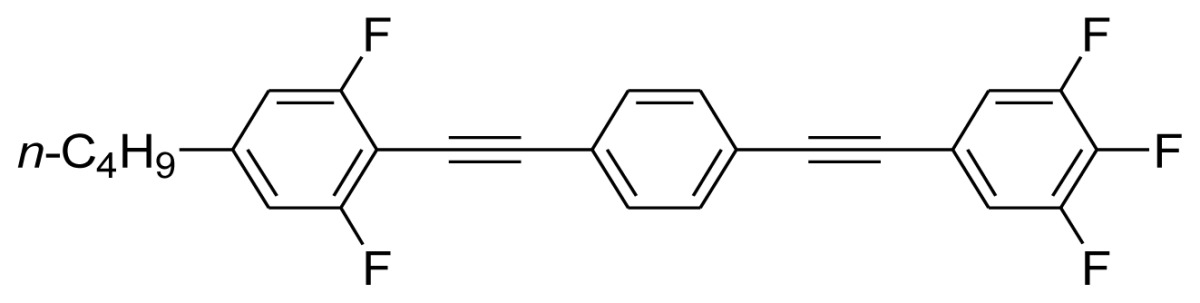	125.2	73.6	c	–
**16**	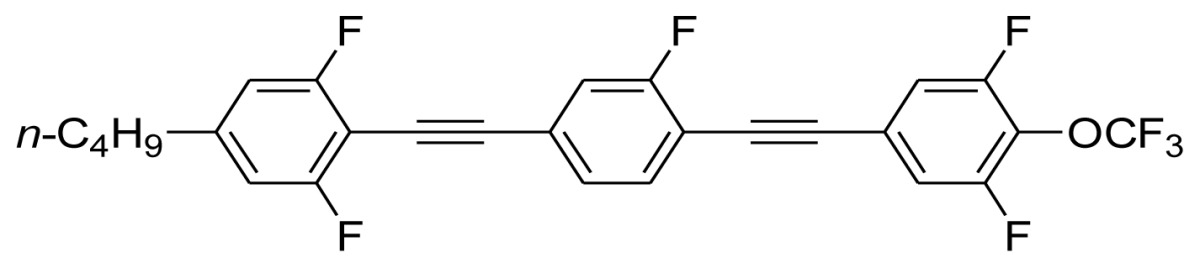	147.8	45.7	–	–
**17**	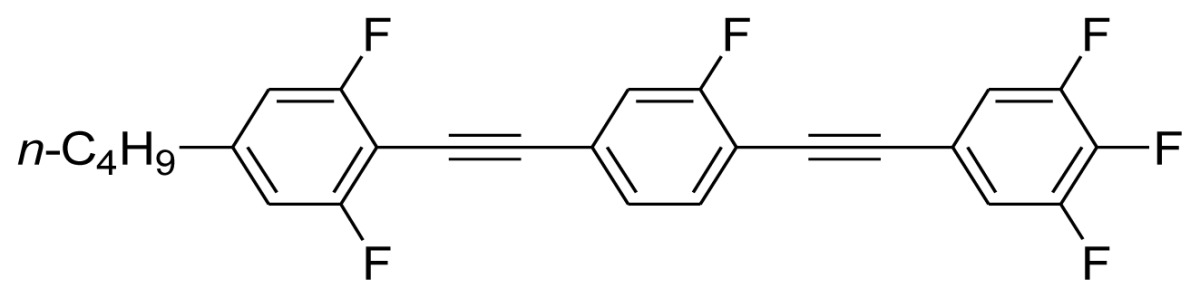	136.2	55.4	–	–
**18**	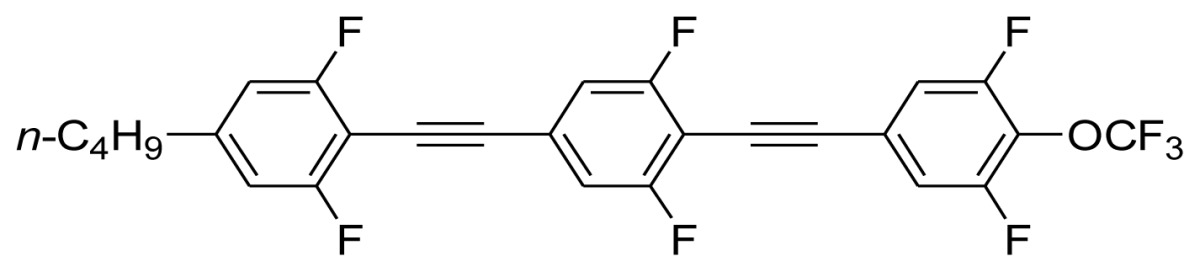	178.3	64.6	–	–
**19**	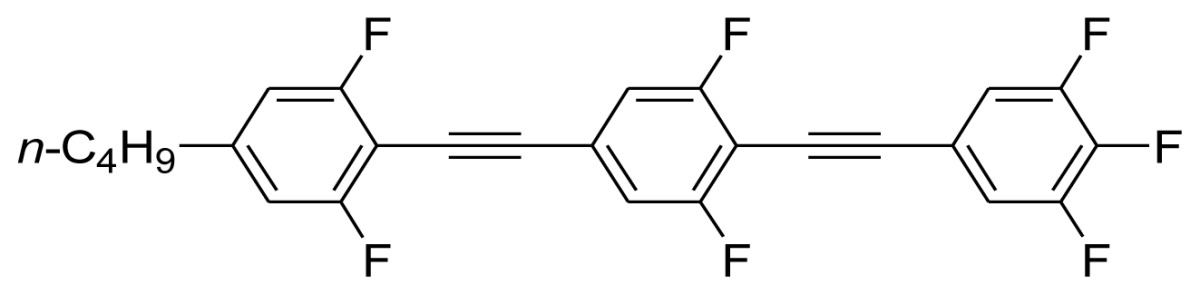	170.8	55.3	–	–
**20**	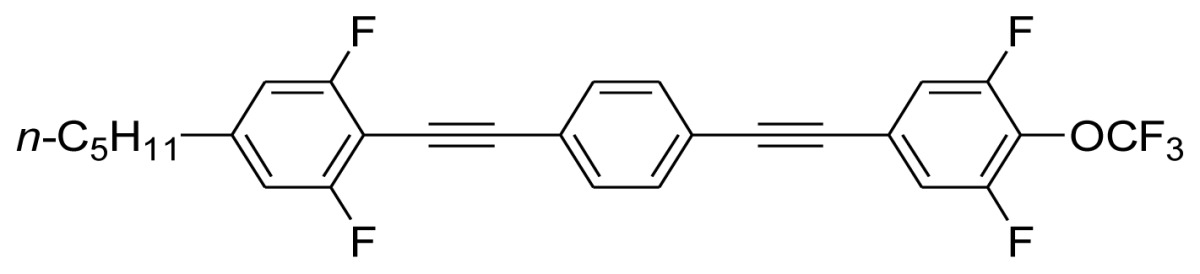	131.3	59.7	144.3	0.81
**21**	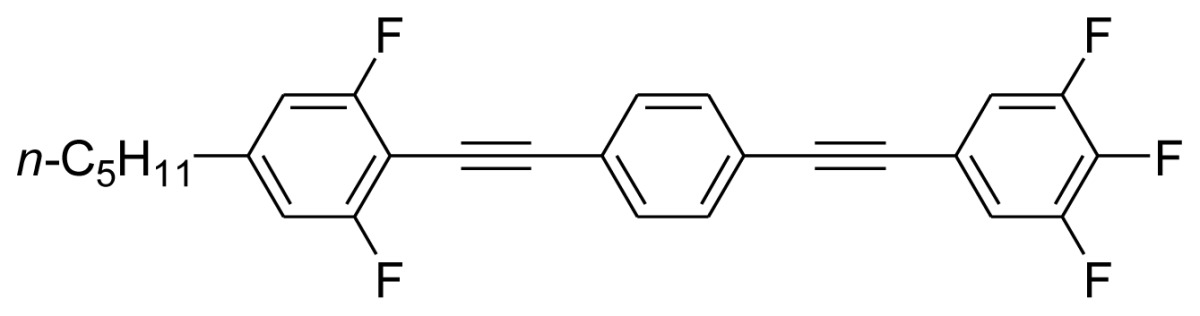	107.7	62.5	122.3	0.29
**22**	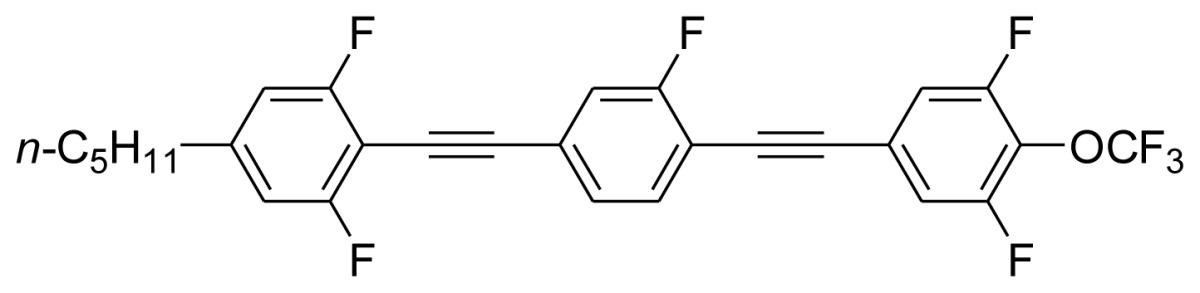	131.3	41.2	–	–
**23**	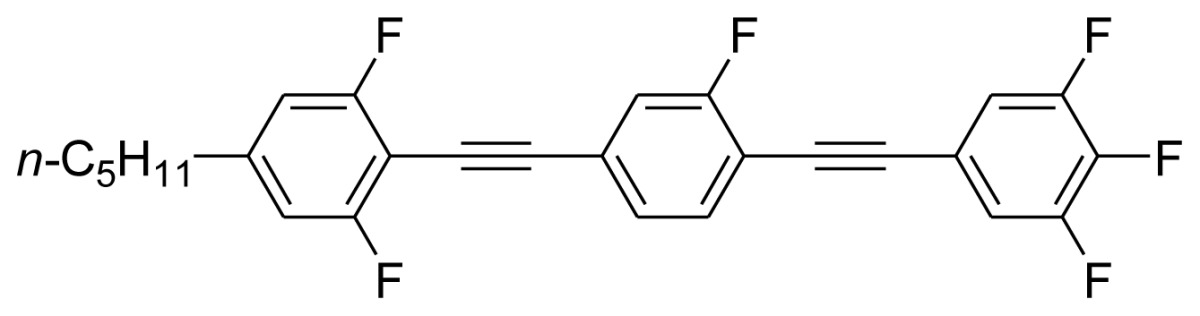	126.0	51.7	–	–
**24**	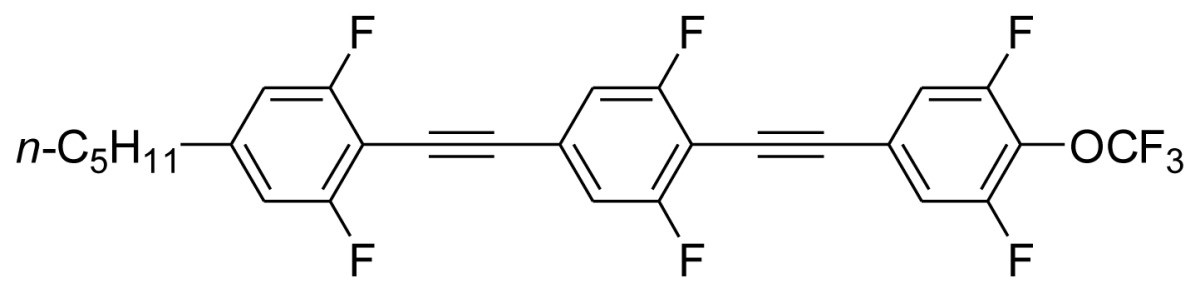	171.1	66.8	–	–
**25**	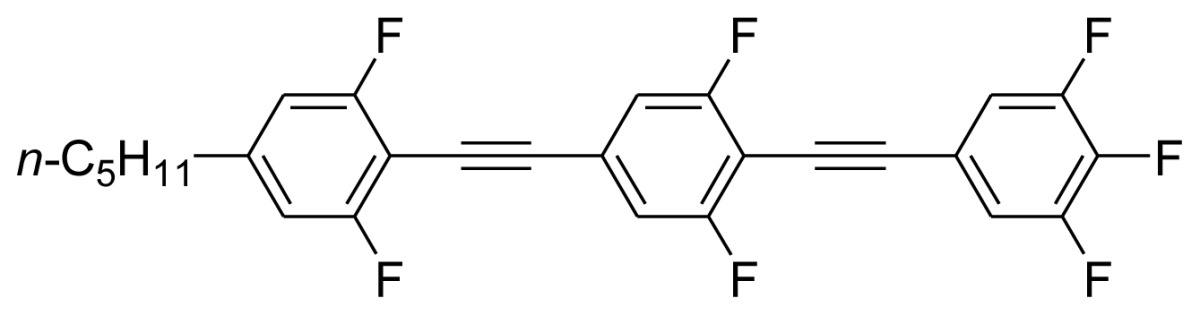	158.7	66.0	–	–

a The nematic phase was only observed during cooling in the temperature range 118.3 to 89.3 °C, and iso-*N* 118.3 °C (Δ*H* = 0.25 J/g), N-Cr 89.3 °C (Δ*H* = 60.4 J/g);

b The nematic phase was only observed during cooling in the temperature range 139.6 to 121.4 °C, and iso-*N* 139.6 °C (Δ*H* = 0.71 J/g), N-Sm 121.4 °C (Δ*H* = 0.61 J/g), Sm-Cr 113.8 °C (Δ*H* = 60.27 J/g);

c The nematic phase was only observed during cooling in the temperature range 107.0 to 71.4 °C, and iso-*N* 107.0 °C (Δ*H* = 0.16 J/g), N-Cr 71.4 °C (Δ*H* = 59.1 J/g).

**Table 2.** Δ*n* values of BPEBs.

BPEB	*n*_e_	*n*_o_	Δ*n*
**1**	2.027	1.531	0.496
**2**	1.987	1.511	0.476
**3**	1.947	1.511	0.436
**4**	2.007	1.511	0.496
**5**	2.103	1.521	0.582
**8**	1.837	1.491	0.346
**9**	1.867	1.521	0.346
**14**	1.797	1.468	0.329
**15**	1.867	1.521	0.346
**20**	1.807	1.491	0.316
**21**	1.867	1.511	0.356

*n*_e_: Extraordinary refraction index; *n*_o_: Ordinary refraction index.

**Table 3.** Δɛ values of BPEBs.

BPEB	ɛ_//_	ɛ_⊥_	Δɛ
**1**	8.9	3.4	5.5
**2**	8.0	3.0	5.0
**3**	7.2	3.0	4.2
**4**	8.1	3.0	5.1
**5**	7.3	3.2	4.1
**8**	29.5	5.5	24.0
**9**	26.1	5.3	20.8
**14**	29.5	6.0	23.5
**15**	26.4	5.4	21.0
**20**	26.1	5.2	20.9
**21**	25.0	5.0	20.0

ɛ_//_: Parallel dielectric constant; ɛ_⊥_: Vertical dielectric constant.
