# Supplementary material for: Synthesis of 1,4-Bis(phenylethynyl)benzenes and Their Application as Blue Phase Liquid Crystal Composition
Source: Int J Mol Sci. 2013 Nov 25;14(12):23257–73. doi: 10.3390/ijms141223257 (PMC3876042; doi:10.3390/ijms141223257)
Supplement: Supplementary file 1 [file ijms-14-23257-s001.pdf]

Supplementary Information

Scheme S1. Preparation of 2,6-difluoro-4-*n*-propylphenyl acetylene (**12c**).

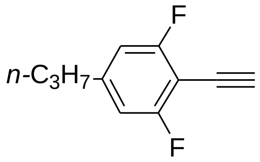

Scheme S2. Preparation of [2,6-difluoro-4-(2',6'-difluoro-4'-*n*-propylphenyl)ethynyl]phenyl acetylene (**12g**).

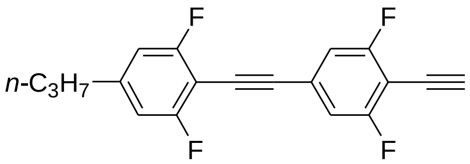

Scheme S3. Preparation of 1-[(2',6'-difluoro-4'-*n*-propylphenyl)ethynyl]-4-[(3",5"-difluoro-4"-trifluoro-methoxy-phenyl)ethynyl]-3,5-difluorobenzene (**BPEB 12**).

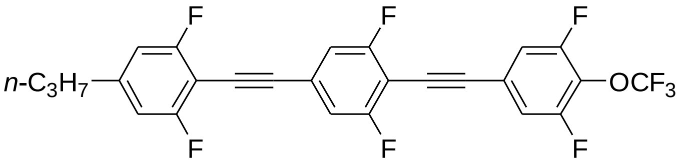

© 2013 by the authors; licensee MDPI, Basel, Switzerland. This article is an open access article distributed under the terms and conditions of the Creative Commons Attribution license (http://creativecommons.org/licenses/by/3.0/).
